# Microstructures and Photodegradation Performance toward Methylene Orange of Sputtering-Assisted Decoration of ZnFe_2_O_4_ Crystallites onto TiO_2_ Nanorods

**DOI:** 10.3390/nano9020205

**Published:** 2019-02-05

**Authors:** Yuan-Chang Liang, Yen-Chen Liu

**Affiliations:** Institute of Materials Engineering, National Taiwan Ocean University, Keelung 20224, Taiwan; sky549144@gmail.com

**Keywords:** sputtering, crystallite, microstructure, photodegradation

## Abstract

In this study, TiO_2_–ZnFe_2_O_4_ (ZFO) core-shell nanorods with various ZFO crystallite thicknesses were synthesized through sputtering-deposited ZFO thin films onto the surfaces of TiO_2_ nanorods. By coupling the ZFO narrow bandgap oxide with TiO_2_, an enhanced photodegradation efficiency of methylene orange under irradiation was achieved. Structural analyses revealed that ZFO crystallites fully covered the surfaces of the TiO_2_ nanorods. The sputtering-deposited ZFO crystallites on the head region of the composite nanorods were markedly thicker than those covering the lateral region of the composite nanorods. The coverage of ZFO crystallites on the TiO_2_ nanorods led to an improved light harvesting, a decrease in the hole–electron recombination rate, as well as the enhanced photodegradation activity of the TiO_2_–ZFO heterostructures under irradiation. The optimized ZFO thickness on the head region of the composite nanorods was approximately 43 nm on average and that at the lateral region of the composite nanorods was 15 nm, which exhibited superior photodegradation ability to methylene orange and retained a stable photodegradation efficiency of approximately 97% after cycling tests. The results herein demonstrate that sputtering deposition of ZFO crystallite with tunable thickness is a promising approach to designing TiO_2_–ZFO composite nanorods with various ZFO coverage sizes and to adjust their photodegradation ability toward organic dyes.

## 1. Introduction

Core-shell nanocomposites exhibit tunable surface, optical, and electronic properties, thus providing a new approach to design the nanomaterials for photodegrading pollutants and various sensing devices [1,2]. TiO_2_ with various morphologies have been developed to use as photocatalyst [3,4]. However, TiO_2_ has an ultraviolet band gap energy. Many efforts have been made in increasing the light harvesting ability of TiO_2_. Noticeable progress has been shown in developing TiO_2_-based core-shell photocatalysts that can work under visible light, and therefore, provide improved photodegradation ability toward organic pollutants [5,6].

Among various visible light sensitizers, ZnFe_2_O_4_ (ZFO) is a semiconductor with a band gap of 1.9–2.7 eV. It is sensitive to visible light and has good chemical stability under irradiation [7]. Moreover, the fabrication of type II heterostructures using TiO_2_ as a matrix could be realized by incorporating ZFO to form a composite structure. This provides a feasible approach to enhance photogenerated charge separation and take one-dimensional (1D) core−shell nanocomposites at the same time. Therefore, constructing TiO_2_–ZFO core-shell heterostructures are promising for applications of degradation toward organic pollutants under illumination. Several TiO_2_–ZFO composites with various morphologies for improving photodegradation ability toward organic pollutants have been reported. For examples, TiO_2_–ZFO composite nanoparticles have been shown to be more effective in photodegradation of organic pollutants than the pure TiO_2_ and ZFO nanoparticles [8]. Sponge-like TiO_2_–ZFO composites derived from a solution combustion method have exhibited superior photodegradation ability toward methylene blue than their single counterparts [9]. The reports on 1D TiO_2_–ZFO core-shell heterostructures are still limited in number. Moreover, most of the literature is focused on chemical solution synthesis of ZFO for integrating in TiO_2_ to form a heterogeneous photocatalyst. The reports on the combinational synthesis method incorporating a physical method, i.e., sputtering, are limited in number. Sputtering is advantageous to provide precise microstructure and composition controls of the oxide thin films and this technique is compatible for the industrial semiconductor process [10,11]. The sputtering is feasible to form ZFO shell layers with different sizes on TiO_2_ nanorods with tunable photodegradation efficiency toward organic pollutants. Therefore, constructing 1D TiO_2_–ZFO core-shell nanorods via sputtering-assisted ZFO crystallite growth provides an ideal approach to manipulate the photodegradation ability of the composite nanorods. In this study, the sputtering deposited ZFO with different sizes are decorated on the surfaces of TiO_2_ nanorods for synthesizing various TiO_2_–ZFO core-shell nanorods. The effects of the ZFO crystallite sizes on microstructure, optical, electrical, and photodegradation ability of the TiO_2_–ZFO core-shell nanorods are investigated herein. An optimal sputtering deposited ZFO shell layer condition for synthesizing the TiO_2_–ZFO nanorods with visible photodegradation efficiency toward methylene orange is reposted.

## 2. Materials and Methods

TiO_2_ nanorods were coated with ZFO thin films with various layer thicknesses. The TiO_2_ nanorods were grown on F-doped SnO_2_ glass substrates using a hydrothermal method. The detailed parameters of hydrothermally derived TiO_2_ nanorods are reported elsewhere [1]. Furthermore, TiO_2_–ZFO composite nanorods were fabricated by sputtering ZFO thin films onto the surfaces of the TiO_2_ nanorod templates. Radio-frequency magnetron sputtering was used to prepare ZFO thin films. The ZFO thin films were grown in mixed Ar/O_2_ ambient with a ratio of 2:1 at 470 °C. The working pressure in the sputtering chamber was maintained at 1.33 Pa. Moreover, the sputtering power of ZFO target was maintained at 100 W. The TiO_2_–ZFO composite nanorods with three different ZFO layer sizes were fabricated by controlling the deposition duration at 45, 60, and 75 min, which corresponded to the samples: TiO_2_–ZFO-1, TiO_2_–ZFO-2, and TiO_2_–ZFO-3, respectively, in this study.

Crystallographic features of the composite nanorods were investigated by X-ray diffraction (XRD; D2 PHASER, Bruker, Karlsruhe, Germany). The surface features of the various nanorods were evaluated by scanning electron microscopy (SEM; S-4800, Hitachi, Tokyo, Japan). High-resolution transmission electron microscopy (HRTEM; JEM-2100F, JEOL Tokyo, Japan) was used to investigate the detailed microstructures of the TiO_2_–ZFO composite nanorods. The attached energy dispersive X-ray spectroscopy (EDS) was used to investigate the composition of the nanorods. A UV-Vis spectrophotometer was used to investigate the transmittance and reflectance spectra of the samples (V750, Jasco, Tokyo, Japan). The elemental binding status of the samples was analyzed by X-ray photoelectron spectroscopy (XPS; ULVAC-PHI XPS, ULVAC, Chigasaki, Japan).

Light irradiation excited from a 100 W Xe arc lamp was used for illumination during the photoactivate measurements. The silver contact electrodes were used for photo-response measurements and the electric measurements were conducted under 5 V. The methylene orange (MO) aqueous solution with a concentration of 5 × 10^−5^ M was used for photodegradation tests. Photodegradation tests of MO solution in the presence of various composite nanorods are performed by comparing the intensity change of the absorbance spectra of the MO solution under illumination with varying durations. The samples for photodegradation tests have a fixed area of 18 mm × 10 mm.

## 3. Results

**Figure 1:**
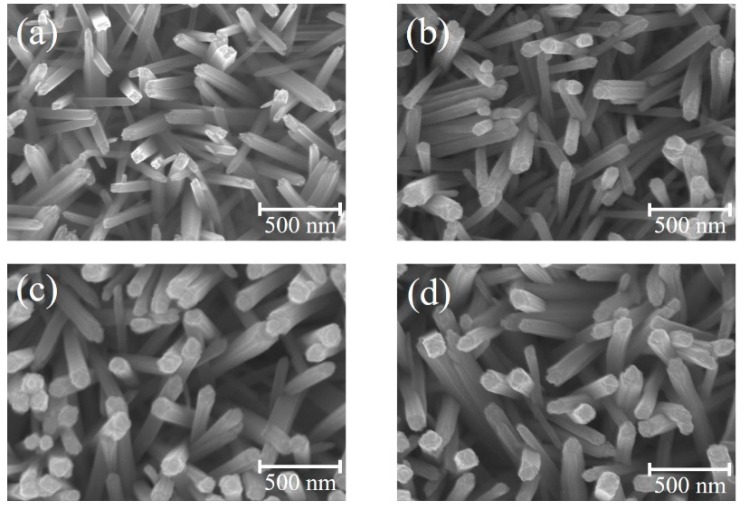
SEM images of various nanorod samples: (**a**) TiO_2_; (**b**) TiO_2_–ZFO-1; (**c**) TiO_2_–ZFO-2; (**d**) TiO_2_–ZFO-3.

Figure 1a–d show SEM images of the TiO_2_ nanorods before and after coating ZFO shell layers with various thicknesses. Figure 1a reveals the presence of many cross-sectional square-shaped TiO_2_ nanorods with an average diameter of 75 nm. Moreover, the TiO_2_ nanorods had an average length of approximately 1.1 um from the cross-sectional image (not shown herein). The smooth surface of the TiO_2_ nanorods can be observed. Notably, Figure 1b–d show the morphologies of the TiO_2_ nanorods after coating ZFO shell layers with various deposition durations is similar to that as shown in Figure 1a. This reveals that the coverage layer thickness of ZFO on the surfaces of the TiO_2_ nanorods are thin and resulted in the fact that the TiO_2_–ZFO composite nanorods still maintained their free-standing feature over the substrates. After the sputtering deposition of ZFO onto the surfaces of TiO_2_ nanorods, the surface feature of TiO_2_ nanorods changed from the smooth surface to the surface composed of many bulges on it. The ZFO was homogeneously loaded onto the surface of TiO_2_ nanorods forming a composite structure. The diameter variation among the various TiO_2_–ZFO composite nanorods with various ZFO sputtering durations was not clearly distinguished from the SEM images herein; this might be associated with the nano-scaled ZFO shell layers on the TiO_2_ surface. To further confirm the ZFO shell layer thickness variation among the various TiO_2_–ZFO samples, TEM investigations were further conducted in this study.

**Figure 2:**
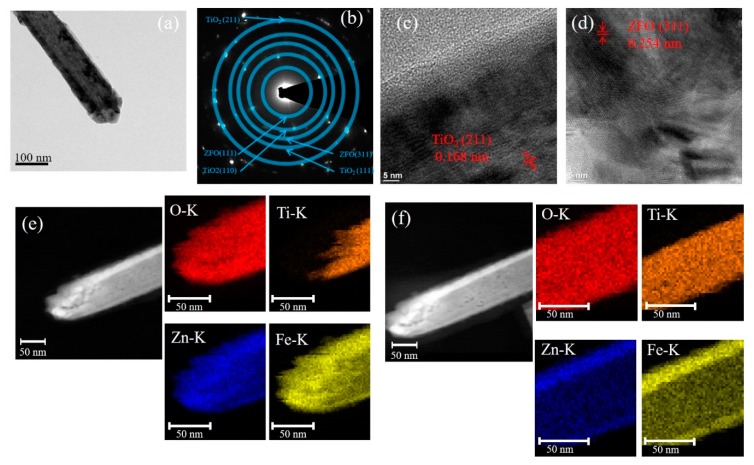
TEM analyses of TiO_2_–ZFO-1 nanorods: (**a**) low-magnification micrograph of the single nanorod; (**b**) selected area electron diffraction (SAED) pattern; (**c**,**d**) high-resolution TEM (HRTEM) micrographs in the lateral and head regions of the nanorod, respectively; (**e**) Zn, Fe, Ti, and O mapping micrographs in the head region of the nanorod; (**f**) Zn, Fe, Ti, and O mapping micrographs in the lateral region of the nanorod.

Figure 2a presents a representative low-magnification TEM micrograph of the TiO_2_–ZFO-1 composite nanorod. The selected area electron diffraction (SAED) pattern taken from several composite nanorods was shown in Figure 2b and it exhibited clear diffraction spots arranged in circles. The centric SAED pattern indicates the co-existence of the ZFO and TiO_2_ phases. Moreover, the TiO_2_–ZFO-1 composite nanorods were in polycrystalline feature. The high-resolution TEM (HRTEM) micrographs from the head and lateral regions of the composite nanorod in Figure 2a are displayed in Figure 2c,d, respectively. The lattice spacing of 0.168 nm for the inner region and 0.254 nm for the outer region of the composite nanorod in the HRTEM images corresponded to the crystallographic plane of (211) for the rutile TiO_2_ and the (311) plane of the cubic ZFO, respectively. Notably, the head region of the composite nanorod consisted of several distinct, large ZFO crystallites and the lateral region of the composite nanorod composed of ZFO in a thin-layered structure. The elemental mapping images of Ti, O, Zn, and Fe from the head and body regions of the TiO_2_–ZFO-1nanorod are demonstrated in Figure 2e,f, respectively. The Ti element was located inside the composite nanorod, while the Zn and Fe elements enclose the rod body, which is definitive proof for the successful synthesis of the core-shell TiO_2_–ZFO nanorod. Notably, the thickness of the ZFO at the coverage region of the composite nanorod’s head is markedly thicker than that at the lateral region of the composite nanorod. The elemental dispersive region size of Zn and Fe at the head region of the composite nanorod is approximately 43 nm on average, and at the lateral region of the composite rod it is only 15 nm. This is due to the fact that the head region of free-standing TiO_2_ nanorods will encounter more sputtering deposited ZFO adatoms than those of the lateral region of the nanorods because of the one-dimensional geometry effect of the TiO_2_ nanorods. The elemental mapping analysis demonstrated that the ZFO was continuously decorated on the TiO_2_ nanorod.

**Figure 3:**
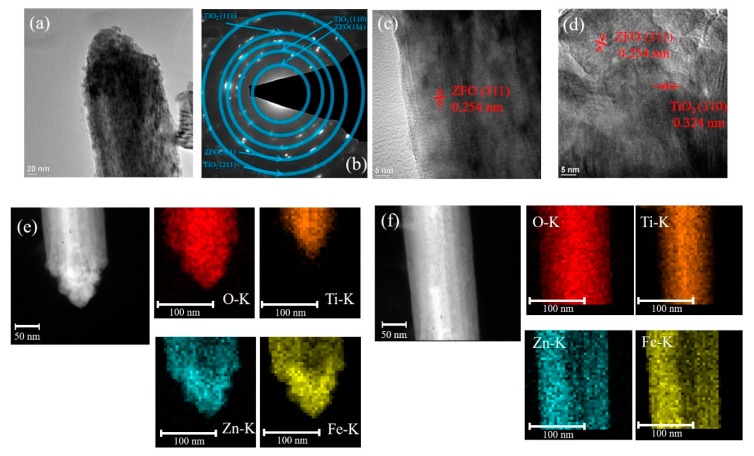
TEM analyses of TiO_2_–ZFO-2 nanorods: (**a**) low-magnification TEM micrograph of the single nanorod; (**b**) SAED pattern; (**c**,**d**) HRTEM images taken from the lateral and head regions of the nanorod, respectively; (**e**) Zn, Fe, Ti, and O mapping micrographs in head region of the nanorod; (**f**) Zn, Fe, Ti, and O mapping micrographs in the lateral region of the nanorod.

Figure 3a presents the low-magnification TEM image of the single TiO_2_–ZFO-2 nanorod. Figure 3b demonstrates the SAED pattern taken from several TiO_2_–ZFO-2 composite nanorods. The clear diffraction spots in centric rings reveals crystalline feature of the TiO_2_–ZFO composite nanorods. The HR images in Figure 3c,d demonstrates the local lattice fringes of the ZFO and TiO_2_ structures taken from the head and lateral regions of the composite nanorod. Similar to the analysis in Figure 2e,f, the coverage thickness of ZFO crystallite is different in the head and body regions of the composite nanorod. The elemental dispersive region size of Zn and Fe at the head region of the TiO_2_–ZFO-2 composite nanorod was approximately 63 nm and at the lateral region of the composite nanorod it was 30 nm (Figure 3e,f).

**Figure 4:**
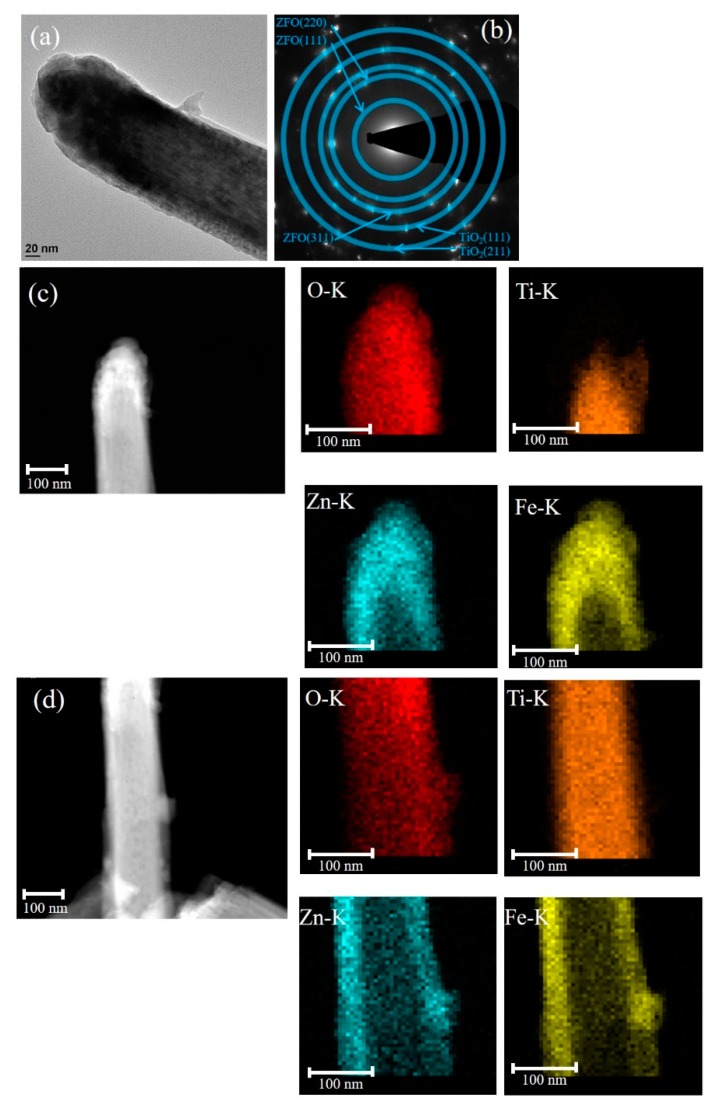
Analyses of TiO_2_–ZFO-3 nanorods: (**a**) low-magnification TEM image of the single nanorod; (**b**) SAED pattern; (**c**) Zn, Fe, Ti, and O mapping micrographs in the head region of the nanorod; (**d**) Zn, Fe, Ti, and O mapping micrographs in the lateral region of the nanorod.

The low-magnification TEM image of the TiO_2_–ZFO-3 nanorod is shown in Figure 4a. The morphology of the head region of the composite nanorod became rounder in comparison with the other composite nanorods prepared at a shorter ZFO sputtering duration. Furthermore, the SAED pattern with distinct spots arranged in centric rings in Figure 4b exhibits the crystalline feature of the TiO_2_–ZFO-3 composite nanorods prepared with the ZFO sputtering duration of 60 min. Notably, the clear local HR images taken from Figure 4a are not available herein because a thicker ZFO covered the TiO_2_ nanorod. The elemental dispersive region size of the Zn and Fe at the head region of the composite nanorod was evaluated to be approximately 97 nm on average and that at the lateral region of the composite, the nanorod was 45 nm from the elemental mapping images in Figure 4c,d. Notably, the increment of the ZFO thickness in the head region was substantially larger than that in the lateral region of the composite nanorod for the prolonged sputtering deposition of ZFO onto the free-standing TiO_2_ nanorods in this study. Nerveless, the ZFO shell layer was fully covered onto the TiO_2_ to form a core-shell composite structure with various ZFO sputtering durations herein.

**Figure 5:**
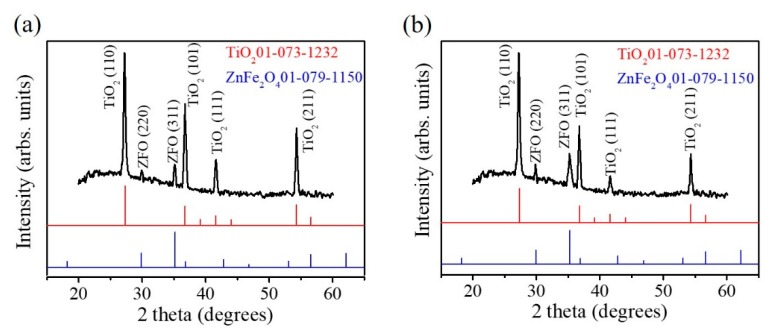
XRD patterns: (**a**) TiO_2_–ZFO-2 nanorods; (**b**) TiO_2_–ZFO-3 nanorods.

The XRD patterns of the TiO_2_–ZFO-2 and TiO_2_–ZFO-3 nanorods are shown in Figure 5a,b to demonstrate the crystal structure of the TiO_2_–ZFO composite nanorods. Notably, the XRD pattern of the TiO_2_–ZFO-1 nanorods is not shown herein because ultra-thin ZFO crystallite thickness coated onto the TiO_2_ nanorods did not give sufficiently intense Bragg reflections from the ZFO. Figure 5a,b exhibit distinct and sharp Bragg reflections originating from the TiO_2_ nanorods (JCPD 01-073-1232). Moreover, small but differentiable Bragg reflections from the ZFO phase were observed in the XRD pattern (JCPD 01-079-1150), revealing the well composited structure in the nanorods. The XRD results are in agreement with the structural observations from the TEM analyses.

**Figure 6:**
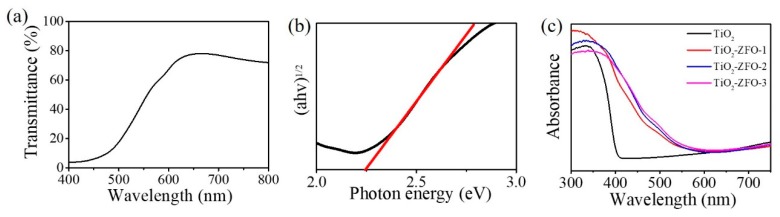
(**a**) Transmittance (%) spectrum of the ZFO thin film. (**b**) Tauc plot of the ZFO thin film. (**c**) Optical absorbance spectra of various nanorods.

The transmittance spectrum of the ZFO thin film is displayed in Figure 6a. The film had a strong absorbance edge in the wavelength of 500~650 nm. Figure 6b shows the estimation of the optical bandgap value of the ZFO thin film by Tauc plot. The optical bandgap value of the ZFO film was 2.25 eV. Furthermore, the UV-Vis diffuse reflectance spectra of the TiO_2_ and various TiO_2_–ZFO composite nanorods are shown in Figure 6c. The absorption edge of TiO_2_ nanorods were located at approximately 410 nm. TiO_2_ nanorods exhibited a strong absorption in the UV range; the estimated bandgap value of the TiO_2_ nanorods was approximately 3.02 eV in this work. After the decoration of the ZFO onto the surfaces of the TiO_2_ nanorods, the absorption edge of the nanorods was substantially red-shifted to the visible light regions, revealing the improved light harvesting ability of the TiO_2_ nanorods via a composite structure. All the TiO_2_–ZFO nanorods with various thicknesses of ZFO shell layer presented an intensive absorption from the UV to the visible light region. Comparatively, the optical absorption edge of the TiO_2_–ZFO-2 and TiO_2_–ZFO-3 nanorods exhibited a larger degree of light harvesting than did the TiO_2_–ZFO-1 nanorods. This might be associated with the ZFO crystallite layer thickness effect. The optical absorption edge analysis revealed that the TiO_2_–ZFO composite nanorods could absorb more photons than that of the TiO_2_ nanorods under solar light irradiation and these composite nanorods are beneficial for photodegrading organic dyes applications.

**Figure 7:**
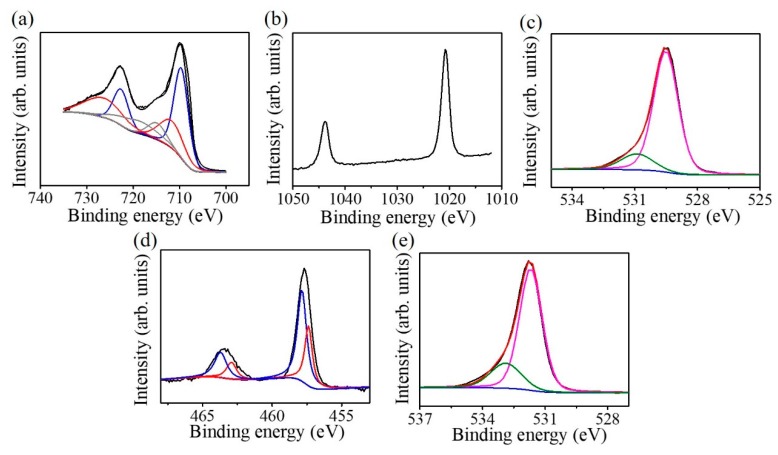
XPS spectra of the ZFO thin film: (**a**) Fe2p; (**b**) Zn2p; (**c**) O1s. XPS spectra of the TiO_2_ nanorods: (**d**) Ti2p; (**e**) O1s.

Figure 7a presents the Fe 2p XPS spectrum of the as-deposited ZFO ultra-thin film; the spectrum can be deconvoluted into six subpeaks. The subpeaks situated at 709.7 and 723.1 eV are ascribed to originate from the oxidation state of Fe^2+^. The subpeaks located at 711.7 and 726.1 eV are attributed to Fe^3+^. The subpeaks situated at 714.8 and 719.1 eV are contributions from the Fe^2+^ and Fe^3+^ satellite signals, respectively [12]. The intense peaks of the binding contribution of 711.7 and 726.1 eV demonstrated that Fe in the ZFO crystallites is mainly in a trivalent state. However, the iron binding energy analysis of the ZFO revealed the existence of a small quantity of Fe^+2^ in the crystal surface. The Zn 2p XPS spectrum is shown in Figure 7b. The peaks located respectively at 1044.1 and 1021.1 eV are from Zn 2p_1/2_ and Zn 2p_3/2_; these binding energies of the Zn 2p imply the existence of Zn^2+^ in the ZFO film [13]. The O 1s XPS spectrum of the ZFO film is shown in Figure 7c. The fitted subpeaks situated at 530.8 eV are associated with the existence of oxygen vacancies in the oxide film. The peak situated at 529.5 eV corresponded to lattice oxygen bonds in the ZFO film. The XPS result demonstrated that the Zn/Fe atomic ratio is 0.49 which is similar to the stoichiometric Zn/Fe composition ratio of the ZFO. However, the oxygen content slightly deviated from the stoichiometry and is approximately 3.5. The existence of oxygen vacancies in the ZFO crystal might account for the observed mixed iron valence states from the Fe 2p signal. Figure 7d displays the Ti 2p XPS spectrum of the TiO_2_ nanorods. The peak was deconvoluted into four subpeaks, which are located at the binding energy regions of approximately 458.1 eV, corresponding to Ti^4+^2p_3/2_, at 463.3 eV, corresponding to Ti^4+^2p_1/2_, at 457.1 eV, corresponding to Ti^3+^ 2p_3/2_, and at 462.8 eV, corresponding to Ti^3+^2p_1/2_. The existence of the Ti^4+^ and Ti^3+^ in the nanorods revealed the presence of oxygen vacancies in the TiO_2_ nanorods. The O1s spectrum of the TiO_2_ nanorods is shown in Figure 7e. The deconvoluted subpeak with a relatively high intensity located at approximately 531.2 eV is ascribed to lattice oxygen of TiO_2_; that with a relatively lower intensity located at approximately 532.3 originated from the oxygen-deficient defects in the TiO_2_. The asymmetric O1s XPS spectrum in Figure 7e, indicates that oxygen vacancies are present on the TiO_2_ surface and this supports the observation of the mixed titanium valence states in the TiO_2_ [14].

**Figure 8:**
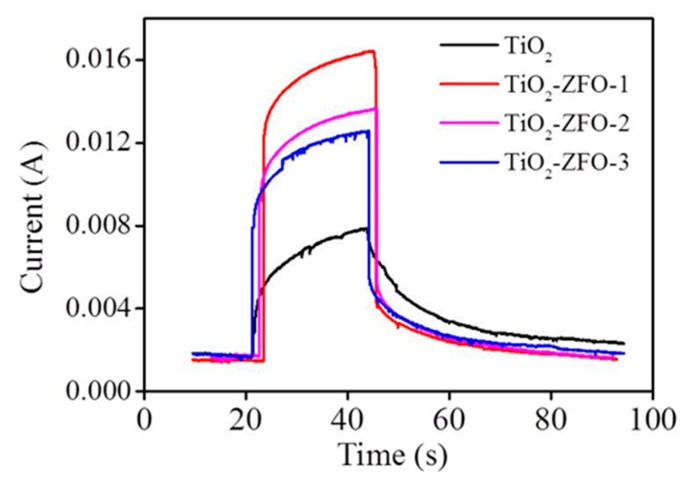
Photocurrent vs. time cures of various nanorods.

Figure 8 shows the current vs. time curves of the TiO_2_ and various TiO_2_–ZFO nanorods with and without irradiation. Comparatively, the measured current value is low for all the nanorods at 5V under dark conditions. The dark current of the various nanorods is in the range of 9 × 10^−4^~1.41 × 10^−3^A. The photocurrent of the nanorods is generated upon light illumination. The photocurrent of the TiO_2_ nanorods is approximately 7.9 × 10^−3^A under irradiation. Moreover, a larger increment of the current value was observed for the composite nanorods under irradiation. The photocurrent values reached 1.32 × 10^−2^A and 1.2 × 10^−2^ for the TiO_2_–ZFO-2 and TiO_2_–ZFO-3 nanorods, respectively. Moreover, the highest photocurrent value of approximately 1.72 × 10^−2^A was observed for the TiO_2_–ZFO-1 nanorods. A substantial increase of the photocurrent of the TiO_2_ coated with the ZFO shell layer was visible. Photo-responses (the ratio of photocurrent to dark current) were 16.8, 13.4, and 12.1, for the TiO_2_–ZFO-1, TiO_2_–ZFO-2, and TiO_2_–ZFO-3 nanorods, respectively. The photo-response of the TiO_2_ nanorods was only 6.4 in this study. The TiO_2_–ZFO-1 nanorods showed a marked increment of photo-response to approximately 2.5 times higher than that of the TiO_2_ nanorods. The photoexcited charges can be responsible for the photocurrent. The intensity of photocurrent is widely used to evaluate the degree of photoexcited charge separation. The rise of the photocurrent means a decreased size of photoexcited charge recombination [15]. The ZFO is a narrow bandgap oxide, which greatly improved the light harvesting ability of the TiO_2_ nanorods. Moreover, the suitable band alignment between the TiO_2_ and ZFO caused the photoexcited electrons in the ZFO to be able to transfer to the conduction band of TiO_2_; moreover, these free electrons flowed through the TiO_2_ nanorods under the bias. [16,17]. Similar to heterogeneous WS_2_–TiO_2_ light detectors, the improved photo-response activity is associated with the type II band structure between the WS_2_ and TiO_2_ [5]. Moreover, the TiO_2_–SnO_2_ nano-heterostructure offers a high charge separation efficiency and a direct pathway for electron transport; therefore, the heterogeneous TiO_2_ detector displays a higher photo-response value than that of TiO_2_ [18]. These examples supported the enhanced photo-response of the TiO_2_–ZFO nanorods than that in the TiO_2_ nanorods herein. An optimal ZFO crystallite size for efficiently improving the photoexcited charge separation in the TiO_2_–ZFO heterostructure was observed for the TiO_2_–ZFO-1 nanorods in this study.

**Figure 9:**
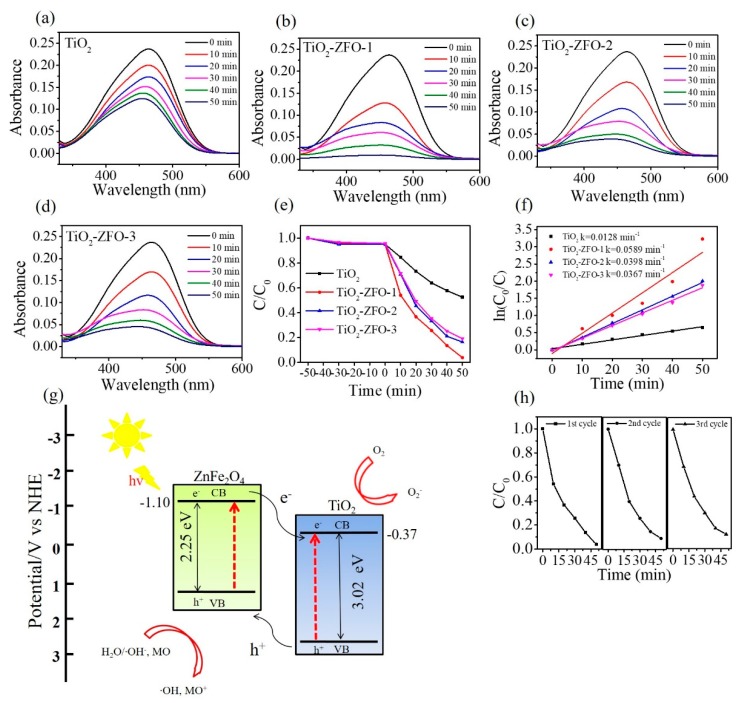
Intensity change of absorbance spectra of MO solution vs. degradation duration containing various nanorods under irradiation: (**a**) pure TiO_2_ nanorods; (**b**) TiO_2_–ZFO-1; (**c**) TiO_2_–ZFO-2; (**d**) TiO_2_–ZFO-3. (**e**) Plot of C/C_o_ vs. irradiation time for MO solution containing various nanorods in dark and illumination conditions. (**f**) Plot of ln(C_o_/C) vs. time. (**g**) Photodegradation mechanism of the TiO_2_–ZFO toward MO dyes. (**h**) Cycling tests.

Figure 9a–d shows the intensity change of absorbance spectra of the MO solution containing various nanorod samples with different irradiation durations. The distinct feature at 464 nm is associated with the monomeric MO. The intensity of the absorbance peaks of the MO solution containing various nanorod samples decreased with the irradiation duration, revealing the photocatalytic reaction between the nanorods and the MO solution under irradiation. A series of contrast experiments were carried out and the photodegradation ratios (C/C_o_) of the MO solution containing various nanorod samples are summarized in Figure 9e. In this study, the C_o_ is the initial concentration of the MO solution without irradiation and C is the concentration of the MO solution with various irradiation times. Notably, prior to illumination, the MO solution containing various nanorod samples was magnetically stirred in the dark for 50 min. Moreover, the peak intensity of the absorbance spectra of the MO solution decreased approximately 6% because partial dye molecules might have been adsorbed onto the surfaces of the nanorods for various nanorod samples under the given dark conditions. From Figure 9e, approximately 47.5% MO solution was degraded under irradiation for 50 min using TiO_2_ nanorods as a photocatalyst. The photodegradation degrees of the MO solution were evaluated to approximately 80.6% and 82.3% when using TiO_2_–ZFO-3 and TiO_2_–ZFO-2 nanorods as photocatalysts, respectively. Furthermore, the highest photodegradation degree of the MO solution was observed for the MO solution containing the TiO_2_–ZFO-1 nanorods at the given degradation reaction condition. For comparison, the kinetic model for photodegrading the MO solution is discussed and the apparent first-order rate constant k was used to evaluating the photodegradation efficiency of different nanorod photocatalysts [2,19]. Figure 9f reveals that the plot of ln(C_o_/C) vs. irradiation duration (t) is approximating linear. The apparent first-order rate constant k was calculated to be 0.0128, 0.0589, 0.0398, and 0.0367 min^−1^ for TiO_2_, TiO_2_–ZFO-1, TiO_2_–ZFO-2, and TiO_2_–ZFO-3 nanorods, respectively. When composite nanorods were used as photocatalysts, the photodegradation efficiency of the MO solution accelerated a lot compared with the pure TiO_2_ nanorods. Moreover, after coating the appropriate size of the ZFO shell layer, the photodegradation rate of the MO solution was further improved. The photodegradation test results illustrated that the photodegradation ability of the TiO_2_ nanorods were substantially improved by decorating ZFO onto the surfaces of the TiO_2_ nanorods. This is attributable to the enhanced light harvesting ability of the TiO_2_ nanorods coated with the visible light sensitizer of ZFO. Moreover, the photo-response result demonstrated that the photocurrent of the TiO_2_ nanorods was substantially enhanced through the surface coating of ZFO. The formation of type II band structure between the TiO_2_ and ZFO is important for the improved photodegradation ability. The effective photogenerated charge separation at the heterointerfaces significantly improved the photodegradation performance of composite nanorods toward organic dyes. This is supported by the case that the construction of ZnO–ZnS core-shell nanorods because of suitable type II band alignment at the heterointerface, substantially improves photodegradation efficiency toward methylene blue dyes [20]. Similarity, the ZnO–SnO_2_ and ZnO–Zn_2_SnO_4_ core-shell nanorods also have a type II band alignment between the constituent compounds and they demonstrate superior photodegradation ability than that of ZnO toward rhodamine B dyes [13]. Figure 9g illustrates the band alignment structure between the TiO_2_ and ZFO and the photodegradation mechanism of the TiO_2_–ZFO composite nanorods. The conduction band (CB) and valence band (VB) positions of the TiO_2_ are determined at −0.37 and 2.65 eV (vs. NHE), respectively [16]. The CB and VB of ZFO are at −1.10 and 1.15 eV (vs. NHE), respectively [17]. Under irradiation, the photogenerated holes in TiO_2_ will migrate to the ZFO. Moreover, the photogenerated electrons in ZFO could migrate to the TiO_2_. This process could improve the photogenerated charge separation efficiency and decline greatly the rate of charge recombination. Therefore, the formation of TiO_2_–ZFO heterojunction structure led to the improvement of photodegradation efficiency of the composite nanorods. The possible photodegradation reactions of the TiO_2_–ZFO composite nanorods with the MO dyes under irradiation are described as follows:

ZFO + *hv* → ZFO (e^−^) + ZFO (h^+^) (1)

TiO_2_ + *hv* → TiO_2_ (e^−^) + TiO_2_ (h^+^) (2)

ZFO/TiO_2_ → charge separation: ZFO (h^+^) + TiO_2_ (e^−^) (3)

ZFO (h^+^) + OH^−^ → ‧OH (4)

TiO_2_ (e^−^) + O_2_ → ‧O^2^ (5)

‧O^2−^ + H^+^ → ‧OOH (6)

‧OOH + H^+^ + e^−^ → H_2_O_2_ (7)

H_2_O_2_ + e^−^ → ‧OH + –OH (8)

h^+^, ‧O^2−^, ‧OH + MO → degradation products (9)

For a series of TiO_2_–ZFO nanorod samples, the TiO_2_–ZFO-1 exhibited superior photodegradation ability toward MO dyes among the various composite nanorods. This might be associated with the fact that thicker ZFO crystallite sizes on the TiO_2_ nanorods engendered the decreased separation efficiency of photogenerated charges in the composites, therefore leading to a decrease of photodegradation rate of the composite nanorods toward MO dyes. This is supported by the observation of different photocurrent values among the various TiO_2_–ZFO nanorods. The increased ZFO shell crystallite size in the TiO_2_–ZFO composite nanorods although is advantageous to generate a greater number of electron-hole pairs in the ZFO side under the given irradiation; however, an increased travel path for the charges to across the heterointerface from ZFO to TiO_2_ might increase the possibility of recombination of charges. Therefore, the TiO_2_–ZFO-1 nanorods exhibited an optimal ZFO shell size and the highest photodegradation performance among various TiO_2_–ZFO nanorods herein. To further test the stability of the TiO_2_–ZFO-1 photocatalysts, recycling tests were conducted. As shown in Figure 9h, the photodegradation rate of the sample remains at a similar level after three consecutive cycles, implying that the obtained TiO_2_–ZFO heterogeneous photocatalyst has a high stability during photodegradation of the MO dyes.

## 4. Conclusions

TiO_2_–ZFO core-shell nanorods with various ZFO crystallite thicknesses were synthesized through sputtering deposited ZFO thin films onto the surfaces of TiO_2_ nanorods. By controlling the deposition duration of ZFO, various ZFO thicknesses were decorated onto the TiO_2_ nanorods. The structural analyses revealed that crystalline TiO_2_–ZFO heterostructures were formed by combining hydrothermal and sputtering methods. Moreover, the coupling of ZFO with TiO_2_ extended the composite nanorods optical absorbance edge to the visible light region. The heterogeneous TiO_2_–ZFO composite nanorods exhibited a superior photo-response value than that of the TiO_2_ nanorods. The experimental results herein demonstrated that the TiO_2_–ZFO-1 nanorods show an optimal ZFO coverage thickness for the decent light harvesting ability and they exhibited the highest degree of photoexcited charge separation efficiency among the various composite nanorods. This further resulted in the higher photodegradation rate of the MO solution for the TiO_2_–ZFO-1 nanorods under irradiation in this study. The experimental results herein demonstrated that sputtering deposited visible-light sensitizer ZFO crystallites with tunable thickness are a promising approach to design TiO_2_–ZFO core-shell heterostructures with optimal photodegradation ability toward organic dyes.
